# Transcriptional regulators and alterations that drive melanoma initiation and progression

**DOI:** 10.1038/s41388-020-01490-x

**Published:** 2020-10-06

**Authors:** Romi Gupta, Radoslav Janostiak, Narendra Wajapeyee

**Affiliations:** 1 Department of Biochemistry and Molecular Genetics, University of Alabama at Birmingham, Birmingham, AL USA; 2 Institute for Research in Biomedicine (IRB Barcelona), The Barcelona Institute of Science and Technology, 08028 Barcelona, Spain

**Keywords:** Cancer genetics, Epigenetics

## Abstract

Although melanoma is the least frequent type of skin cancer, it accounts for the majority of skin cancer-related deaths. Large-scale sequencing efforts have led to the classification of melanoma into four major subtypes (i.e., BRAF-mutant, NRAS-mutant, NF1-deficient, and triple wild-type). These sequencing studies have also revealed that melanoma genomes are some of the most mutated genomes of all cancers and therefore have a high neoantigen load. These findings have resulted in the development and clinical use of targeted therapies against the oncogenic BRAF→MEK→ERK pathway and immune checkpoint inhibitors for the treatment of metastatic melanoma. Although some patients with metastatic melanoma benefit immensely from these transformative therapies, others either become resistant or do not respond at all. These clinical challenges have intensified the search for new drug targets and drugs that can benefit patients who are either intrinsically resistant or have acquired resistance to targeted therapies and immunotherapies. Numerous signaling pathways and oncogenic drivers can cause changes in mRNA transcription that in turn drive melanoma initiation and progression. Transcriptional regulation of mRNA expression is necessary to maintain cell identity and cellular plasticity via the regulation of transcription factor expression and function, promoter/enhancer activities, chromatin regulators, and three-dimensional genome organization. Transcriptional deregulation can arise due to genetic and/or non-genetic alterations in the genome. Specifically, these deregulated transcriptional programs can become liabilities for melanoma cells due to their acquired dependencies on these programs for survival, which can be harnessed to develop new therapies for melanoma. In this article, we present an overview of the mechanisms that result in the transcriptional deregulation of mRNA expression in melanoma cells and assess how these changes facilitate melanoma initiation and progression. We also describe how these deregulated transcriptional pathways represent new opportunities for the development of unconventional and potentially impactful treatments for metastatic melanoma.

## Introduction

Melanoma accounts for over 132,000 cases each year worldwide. The incidence of melanoma is higher in Western counties than in other parts of the world, with the highest incidences occurring in Australia and New Zealand. Basic research to understand the genetics of melanoma and the subsequent translation of fundamental discoveries into clinical applications has resulted in the development and use of several transformative therapies to treat patients with metastatic melanoma. Among those are therapies that target the BRAF→MEK→ERK pathway, and particularly the use of BRAFV600E and MEK inhibitors to treat BRAF-mutant melanoma. The melanoma genome is highly mutagenized and, therefore, has a high neoantigen load, making melanoma significantly more immunogenic compared with other cancers. Therefore, melanoma has emerged as one of the cancer types that is most responsive to immune checkpoint blockage-based immunotherapies.

Transcriptional regulation of mRNA expression serves as a key determinant of transcriptome and proteome diversity in mammalian cells. Patterns of mRNA expression determine cell identity and allow cells and tissues to acquire specific functional and phenotypic characteristics, which collectively are necessary for whole-body homeostasis to maintain normal physiology and organismal survival. Genetic or epigenetic alterations in cancer cells can hijack transcriptional networks to attenuate tumor-suppressive transcription and promote oncogenic transcription factor expression and pro-oncogenic gene expression signatures. The deregulation of mRNA expression is one of the characteristics of all cancer cells, and most cancer types can be classified solely on the basis of their mRNA expression patterns [1, 2]. Because transcriptional changes in cancer cells enable many cancer hallmarks, cancer cells become dependent on deregulated transcriptional networks for proliferation and survival.

Transcriptome-wide profiling of patient-derived melanoma samples has led to the identification of specific signatures associated with major oncogene subtypes (e.g., BRAF-mutant, NRAS-mutant, NF1-mutant, and triple wild-type) and prognoses [3–5]. More recently, a study of melanoma samples by The Cancer Genome Atlas (TCGA) took a different approach to provide a transcriptome-based classification of melanomas. Based on the gene functions of 1500 discriminatory mRNA transcripts across 329 melanoma samples, the TCGA study identified three melanoma clusters, which were subsequently named the immune subtype, the keratin subtype, and the micropthalmia-associated transcription factor (MITF)-low subtype. The post-accession survival (i.e., survival calculated from the date of biospecimen collection/accession to the date of last follow-up or death) of patients with regionally metastatic tumors was significantly different among the three clusters; patients with the immune subtype had the best survival, whereas those with the keratin subtype had the worst survival. These results suggested that the differences among the transcriptional subtypes are biologically important and that changes in mRNA transcription profiles are important drivers of melanoma initiation and progression.

Melanoma cells can acquire changes in transcriptional pathways and accumulate dysregulated gene expression by a variety of mechanisms. Among these mechanisms are the direct mutations of transcription factors and chromatin regulators or changes in expression that alter their specificity, localization, association with other proteins, and/or promoter occupancy. Similarly, direct mutagenesis of histone proteins and noncoding regulatory genomic regions can create new sites for transcriptional regulators to bind and abolish the old binding sites. In addition, three-dimensional architectural changes can lead to changes in gene expression by altering topologically associated domains (TADs) and long-range DNA/chromatin interactions.

In most cases, transcriptional deregulation benefits melanoma cells by allowing them to proliferate under various stressful conditions and thus spread to distant organs. Therefore, deregulated transcription pathway regulators represent unconventional and largely untapped targets for drug-target discovery and drug development. In this review, we provide an account of key drivers of transcriptional alterations of mRNA expression in melanoma, and discuss how the discovery of these drivers provides new opportunities to develop drugs to treat metastatic melanoma.

## Transcriptional regulators in melanoma

**Fig. 1:**
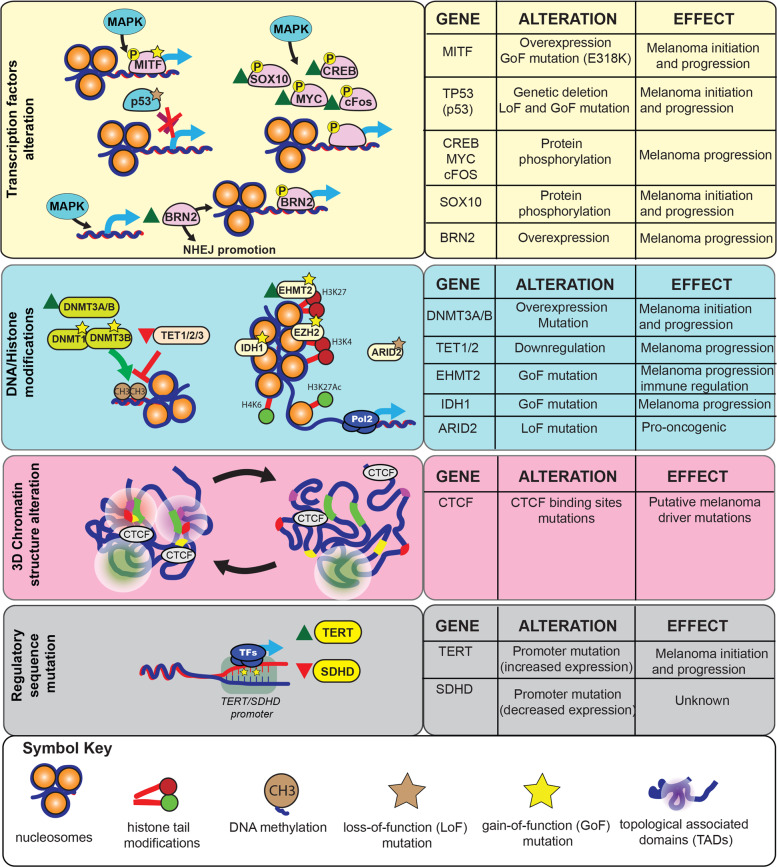
Mechanisms and regulators driving transcriptional deregulation in melanoma. (Left) Transcriptional regulators and changes in long-range DNA interactions that drive transcriptional deregulation in melanoma. (Right) Summary of individual transcriptional regulators and changes in long-range DNA interactions, their alterations in melanoma, and their impact on the initiation and progression of melanoma.

Transcription regulation is a complex biological process that in eukaryotic cells involves a series of regulators, including transcription factors, co-activators, and chromatin regulators. The transcription of coding RNAs (mRNAs) and noncoding RNAs (microRNAs, long noncoding RNAs, transfer RNAs, and ribosomal RNAs) is deregulated in melanoma. In this review, we will focus on transcriptional deregulation of mRNA expression, which can occur via the following four major mechanisms: (1) genetic or non-genetic modulation of transcription factors; (2) genetic or non-genetic modulation of DNA-modification proteins, chromatin regulatory proteins, or histone proteins; (3) genetic or non-genetic modulation of gene regulatory elements; and (4) chromatin conformation changes (Fig. 1). In the next four sections, we use some key examples to describe how these mechanisms drive mRNA transcriptional deregulation in melanoma.

### Genetic and non-genetic modulation of transcription factors

The simplest mechanism by which transcriptional dysregulation occurs in cancer cells is the genetic mutation or non-genetic modulation of transcription factors. An example of a melanoma-driving transcription factor is MITF, which was identified as a lineage-specific oncogene using an integrative approach in which single nucleotide polymorphism (SNP) array data were combined with gene expression analysis of the NCI-60 panel of cell lines [6]. This study showed that MITF is overexpressed in about 20% of metastatic melanomas. MITF is necessary for the survival of normal human melanocytes, and that dependency is maintained in melanoma cells that overexpress MITF [6, 7]. In addition to copy number gains, mutations have also been reported in MITF. In particular, a recurrent E318K mutation in MITF was identified that was associated with gain-of-function activity and shown to predispose individuals with this mutation to familial and sporadic melanoma [8]. This study also noted that the E318K mutation is located within IKXE consensus sites in MITF, which were previously shown to be post-translationally modified by sumoylation, resulting in reduced transcriptional activity [9, 10]. Therefore, this study tested whether the E318K mutation caused the abrogation of MITF sumoylation. Consistent with this hypothesis, not only did the E318K mutant abolish MITF sumoylation, but it also enhanced MITF transcriptional activity and MITF-regulated gene expression [8]. These studies collectively underpin the complex nature of transcription factor expression and the mechanisms involved in regulating post-translational activities that play vital roles in the initiation and progression of melanoma.

Transcription factors can also be regulated as a result of non-genetic changes, such as post-translational modification and/or changes in interacting partners, which in turn govern transcription factor localization, DNA binding, and association with transcriptional co-activator proteins. Consistent with the role of post-translational modification in the modulation of transcription factor function, MITF is post-translationally modified by a number of kinases. For example, c-KIT upregulates MITF expression via MEK/ERK pathway-mediated phosphorylation of MITF at Serine 73 [11]. Similarly, GSK3β and p38 mitogen-activated protein kinase (MAPK) have been shown to phosphorylate MITF at Serine 298 and Serine 307 [12, 13]. These phosphorylation events are important for MITF transcriptional activity and, therefore, for the ability of MITF to activate target-gene expression.

Another transcription factor that is mutated in melanoma is tumor suppressor p53, which is mutated in about 15% of melanomas [14]. Mutations in p53 are typically either in-frame or missense mutations [14]. Mutations in p53 have been shown to prevent p53 from binding to DNA, thereby hampering the ability of p53 to activate its target genes [15]. In addition, mutations in p53 can result in gain-of-function mutations that allow mutant p53 to acquire neomorphic functions that impart oncogene-like, cancer-promoting activities [15]. Additionally, the post-translational modification of p53 can determine which transcriptional targets can be activated by p53 [16]. Similarly, interacting proteins have been shown to modulate p53-mediated transcription. An example is PGC-1α, which interacts with p53 to promote cell survival upon metabolic stress. PGC-1α binding alters the transactivation function of p53, resulting in the preferential activation of genes that regulate cell cycle and metabolism [17].

Another interesting example of a transcription factor with important roles in melanoma is the transcription factor SR-BOX 10 (SOX10), which is characterized by a DNA-binding motif known as the high mobility group (HMG) domain. SOX10 is well known for its role in neural crest and oligodendrocyte development; it becomes upregulated in the pre-migratory cells of the neural crest and is essential for the committed development of neural crest cells into melanocytes [18, 19]. An interesting example of the role of SOX10 in melanoma is its promotion of the formation and maintenance of giant congenital naevi [20]. Giant congenital naevi are pigmented childhood lesions that often have NRAS mutations rather than BRAF mutations; the presence of these lesions also confers an enhanced risk of early onset melanoma [21]. In a mouse model of giant congenital naevi, Sox10 haploinsufficiency was shown to inhibit Nras(Q61K)-driven congenital nevus and melanoma formation [20]. In human cells, the loss of SOX10 was shown to result in reduced CD271-positive, tumor-initiating cells [20]. This study’s findings were also supported by the fact that in human patients, virtually all congenital naevi and melanomas were SOX10 positive [20].

Another example of the role of SOX10 in melanoma is the finding that ERK phosphorylates and regulates SOX10 sumoylation at lysine 55, which is required for the regulation of its transcriptional activity and target selection in BRAF-mutant melanoma. In particular, this study discovered that SOX10 functions as a regulator of the stemness transcription factor FOXD3. This study also showed that the depletion of SOX10 sensitized BRAF-mutant melanoma cells to BRAF inhibitors [22]. Collectively, these studies support a prominent role for SOX10 in the initiation and progression of melanoma.

Similarly, brain-specific homeobox/POU domain protein 2 (BRN2), also known as POU3F2, has been shown to promote melanoma metastasis. Specifically, BRN2 was shown to be a target of the MAPK pathway [23], which promotes melanocyte-specific gene expression and melanoma tumor growth [24, 25]. BRN2 has also been shown to repress MITF expression and mark a distinct subpopulation of MITF-negative melanoma cells. Furthermore, related to its role in promoting melanoma metastasis, BRN2 confers anoikis resistance and promotes metastatic growth after the dissemination of melanoma [26, 27]. A separate study identified a previously undocumented role for BRN2 [28]. This study showed that, independent of its transcription factor function, BRN2 associates with DNA damage response proteins and directly binds PARP1 and Ku70/Ku80. This association promotes Ku-dependent nonhomologous end joining (NHEJ) instead of homologous recombination. To further highlight the importance of this finding, this study documented that BRN2 expression also correlated with a high prevalence of single nucleotide variations in cases of human melanoma. Based on these collective findings, this study suggested that by promoting error-prone NHEJ-based DNA repair, BRN2 contributes to a high mutation burden in melanoma [28]. Overall, this study highlights the important fact that several melanoma-promoting transcription factors, similar to BRN2, might mediate some of their key biological effects independent of their ability to regulate transcription.

The MAPK pathway that is activated in melanoma as a result of mutations in BRAF, NRAS, or NF1 has been shown to regulate the expression, stability, and activity of a number of different melanoma-relevant transcription factors. In particular, sustained MAPK pathway activity can lead to phosphorylation and increased stability of c-FOS [29]. Similarly, MEK→ERK signaling regulates the expression, stability, and activity of a number of other transcription factors such as CREB, FOXO3a, MYC, and c-JUN [30–34]. Collectively, studies on various transcription factors, whose expression and/or function is altered in melanoma, highlight transcription factor dysregulation as a key factor that shapes mRNA transcription in melanoma and promotes the initiation and progression of melanoma.

### Genetic and non-genetic modulation of DNA-modifying proteins, chromatin regulatory proteins, and histones

DNA and chromatin regulators are often mutated or post-translationally modified in melanoma. Two major groups of DNA-modifying proteins have been identified and functionally categorized: DNA methyltransferases (DNMTs) and DNA demethylases. DNMTs catalyze the transfer of a methyl group from S-adenyl methionine to the fifth carbon of cytosine residues to form 5-methylcytosine (5mC) [35]. DNA demethylases have enzymatic activity opposite to that of DNMTs, that is, the stepwise removal of 5mC from DNA in conjunction with other proteins [36].

Among the three major DNMTs, DNMT3A and DNMT3B are commonly overexpressed in melanoma [37, 38]. Additionally, SNPs in DNMT1 (rs2228612, rs2228611, and rs2114724) and DNMT3B (rs406193 and rs2424932) have been shown to affect the clinical course and disease outcome in patients with melanoma [39]. For example, carriers of the rs2228612 genotype of DNMT1 had poorer overall and recurrence-free survival than patients with the wild-type allele [39]. In contrast to DNMTs, the ten eleven translocation (TET) family of DNA demethylases (TET1, TET2, and TET3) are commonly downregulated in melanoma, and melanoma cells accordingly show reduced levels of 5hmC in their DNA compared with melanocytic nevi [40]. Collectively, the studies on DNA modification proteins indicate a potential oncogene-like role for DNMTs and a tumor suppressor-like role for TET proteins in melanoma. Later in this review, we discuss additional studies that support this notion.

Like DNA, histone proteins can undergo post-translational modification. Post-translational changes to histones serve as regulatory signals for transcription. Chromatin regulatory proteins are classified as writers, erasers, or readers based on their activity [41]. Writer proteins post-translationally modify histones. An example is Enhancer of Zest 2 (EZH2), which acts as an H3 lysine 27 methyltransferase (H3K27me3) [42]. Depending on the type of histone modifications, chromatin can either become less compact or more compact, making it permissive or nonpermissive for transcription, respectively. Eraser proteins remove post-translational chemical marks from histones. An example is the Jumanji histone demethylase KDM4A, which functions as a trimethylation-specific demethylase [43]. Reader proteins monitor histone modifications and either activate or repress transcription depending on the state of the histones [44]. An example is the bromodomain-containing proteins, which recognize acetyl-lysine modifications, facilitate chromatin remodeling, and regulate transcription [45].

Several examples of direct alterations of histone writers, erasers, and readers have been identified in melanoma [14]. For example, one TCGA study identified loss-of-function mutations in AT-rich interacting domain 2 (ARID2) and gain-of-function mutations in isocitrate dehydrogenase 1 (IDH1). ARID2 is a subunit of the PBAF chromatin-remodeling complex, which facilitates ligand-dependent transcriptional activation by nuclear receptors [46]. IDH1 is a dimeric cytosolic NADP-dependent isocitrate dehydrogenase that catalyzes the decarboxylation of isocitrate into alpha-ketoglutarate. Mutations in IDH1 have been shown to affect histone and DNA methylation [47, 48]. The frequency of ARID2 mutations among different melanoma genetic subtypes ranged from 2% in triple wild-type melanoma to 29% in NF1-mutant melanoma [14]. By contrast, the frequency of IDH1 mutations was more uniform across melanoma subtypes, ranging from 4% to 9% [14]. Similar to IDH1, EZH2, a member of polycomb repressive complex 2, is another recurrent site of gain-of-function mutations in melanoma, although at a lower frequency [49]. EZH2 mutation (EZH2^Y646^) is observed in 3% of human melanomas, and focal amplification of EZH2 was noted in 15 of 262 (5.7%) cases of melanoma in TCGA [14].

A recent example of histone writer deregulation in melanoma is the histone methyltransferase EHMT2 (also known as G9a). EHMT2 is a histone methyltransferase that methylates histone H3 on lysine 9 (H3K9me2) [50]. One study identified previously unreported recurrent activating mutations (e.g., G1069) in EHMT2, as well as EHMT2 copy number gains in ~26% of human melanomas, which is similar to the pattern of EZH2 gain-of-function mutations [51]. Although histone mutations have been observed in several cancer types, such as head and neck squamous cell carcinoma and glioma, they are rare in melanoma and are typically restricted to acral and desmoplastic subtypes [52]. These findings highlight the need for continued efforts by independent researchers to identify new, potentially targetable chromatin-modified alterations beyond what has been identified by large-scale consortium studies such as TCGA. Overall, studies on proteins that modify DNA and chromatin structure have identified widespread alterations of chromatin regulatory proteins that are expected to directly impact mRNA transcription in melanoma.

### Genetic modulation of gene regulatory elements leading to transcription deregulation

Although cancer genome sequencing studies initially focused on genetic mutations in protein-coding genes, recent studies have looked at noncoding regions and discovered intronic mutations and promoter/enhancer mutations that directly impact the transcription of cancer regulatory genes [53, 54]. An example of such a mutation is the identification of mutations in the telomerase reverse transcriptase (TERT) promoter in melanoma and other cancers. The original quest to analyze the TERT promoter for mutations was driven both by the question of how telomerase is overexpressed in human cancer cells and by the question of whether mutations in noncoding regions play a direct role in driving cancer. Mutations in the TERT promoter remain the most frequent mutations in the transcriptional regulatory regions in melanoma and other types of cancer [54–56]. Hot-spot mutations in the TERT promoter were shown to create a TTCC response element, which is a highly conserved binding site for ETS transcription factors. It is worth mentioning that, in some cases, TERT promoter mutations correlated with increased TERT expression; however, such a correlation was not always observed [57]. Therefore, mutation of the TERT promoter cannot be the sole mechanism by which telomerase expression is increased in cancer.

In addition to TERT promoter mutations, a less well-described mutation in the succinate dehydrogenase complex subunit D (SDHD) promoter has been observed in melanoma [54]. Unlike mutations in the TERT promoter, mutations in the SDHD promoter were found exclusively in melanoma and not in other cancer types. In contrast to TERT mutations that create new ETS-binding sites, mutations in the SDHD promoter disrupt ETS-binding sites [54]. SDHD promoter mutations that abolished ETS-binding sites in melanoma cells resulted in significantly lower SDHD expression compared with that in melanoma cells without SDHD promoter mutations. Furthermore, chromatin immunoprecipitation (ChIP) data from Encyclopedia of DNA elements (ENCODE) revealed a strong positive correlation between the expression levels of SDHD and the ETS transcription factor ELF1 in melanoma cells without SDHD promoter mutations, indicating a possible role of ELF1 in the regulation of SDHD under normal conditions. The studies on mutations in the TERT and SDHD promoters highlight promoter-region mutations as important events that regulate the transcription of cancer regulatory genes by creating or abolishing transcription factor-binding sites.

### Chromatin accessibility, TADs, and long-range chromatin interactions as regulators of mRNA transcription

One of the prerequisites for mRNA transcription is the ability of transcription factors and activator proteins to access open chromatin. The National Institute of Health (NIH) Roadmap Epigenomics Consortium analyzed 111 reference human epigenomes and an additional 16 samples from ENCODE to uncover how epigenetic processes contribute to human biology and disease [58]. One of the major components of the consortium was the Reference Epigenome Mapping Centers, the goal of which was to characterize the epigenomic landscapes of representative primary human tissues and cells. A series of different assays were used to analyze primary tissues and cells, including DNAse I hypersensitivity profiling to map accessible DNA on the human genome. The results showed that enhancers with strong H3K27ac had higher DNA accessibility, lower methylation, and higher transcription factor binding compared with enhancers lacking H3K27ac, indicating that H3K27ac can be used as a predictor of highly transcribed mRNAs.

The chromatin state is an important predictor of the mutational landscape in cancer cells. The Roadmap Epigenomics Consortium performed a landmark study in which they showed that the cell-of-origin chromatin organization shapes the mutational landscape of cancer. Chromatin accessibility and modification in combination with replication timing explained up to 86% of the spatial variance in mutation rates among cancer genomes [59]. The best predictors of local somatic mutation density in different types of cancer were epigenomic features derived from the cell types that gave rise to the cancers [59]. In fact, the cell-of-origin chromatin features were much stronger determinants of cancer mutation profiles than the chromatin features of matched cancer cell lines. Moreover, chromatin features were a stronger predictor than gene expression of the mutational density and were able to link 88% of melanoma samples accurately to their cells of origin (melanocytes) [59]. The study went on to show that the DNA sequence of a cancer genome contains information on the identity and epigenomic features of the cells of origin. Eight different cancer types, including melanoma, were included in the study. In the case of melanoma, the mutational density was associated with individual chromatin features specific to melanocytes [59].

More recently, another large-scale study used the assay for transposase-accessible chromatin with high-throughput sequencing (ATAC-seq) to analyze the chromatin accessibility landscape of 410 tumor samples representing 23 different types of primary human tumors, including 13 melanoma samples [60]. The study integrated the ATAC-seq results with DNAse I hypersensitivity sites sequencing of normal tissues from the NIH Roadmap Epigenomics Consortium. In total, 65% of the pan-cancer peaks identified overlapped with previously observed regulatory elements, highlighting the ability of the ATAC-seq-based approach to identify a large number of putative regulatory elements. On average, 16,982 peaks were identified per cancer sample. Transcription-accessible sites were strongly enriched in promoter and enhancer regions, as determined by the overlap of the ATAC-seq-defined regulatory elements with ChIP-seq-defined ChromHMM regulatory states. ChromHMM is a software for learning and characterizing chromatin states, which can integrate multiple chromatin datasets, such as ChIP-seq data for various histone modifications for the discovery of combinatorial and spatial patterns [61]. The samples formed clusters based on patterns of chromatin accessibility. Using a framework called distal binarization, the researchers showed that of the 516,927 pan-cancer distal elements, 203,260 were highly accessible in only a single cluster or a small group of clusters. These cluster-specific peaks were enriched with motifs of transcription factors linked to genes known to be important for cancer and tissue identity, including MITF for melanoma. Motifs associated with POU5F1, DRGX, PHOX2B, and CDX2 were also enriched in the cluster-specific peaks, albeit less strongly than the MITF motifs. Thus, ATAC-seq-based chromatin accessibility alone was able to accurately classify various cancers and cancer subtypes, similar to mRNA expression profiles. Another interesting aspect of the study was the discovery of correlations among promoter-region mutations, chromatin accessibility, and mRNA expression. For example, when combined with genome-wide sequencing data, the results revealed positive correlations between TERT and FGD4 mutations, chromatin accessibility, and mRNA expression levels. Further studies using more melanoma samples will likely provide more insights into the relationships between transcriptional regulators, chromatin accessibility, and drivers of melanoma initiation and progression.

A simplistic view of transcriptional regulation is that it is a locus-specific event with RNA pol II and other transcription factors and machinery assembling to transcribe mRNA. It has become clear, however, that long-range interactions among promoters and enhancers can result in the activation or repression of mRNA expression [62]. Recently, it was shown that the genome is organized into TADs that are several hundred kilobases in size and encompass multiple genes and regulatory elements [63, 64]. TADs are conserved and largely invariant across different cell types [65, 66]. The insulator protein CTCF and cohesin were shown to be involved in the formation and maintenance of TADs [65, 66]. Studies of TAD organization revealed widespread disruption of TAD organization in cancer cells and cancer-specific changes in promoter–enhancer interactions within individual TADs, resulting in altered transcription of mRNAs that are deregulated in cancer and promote tumor initiation and progression [67, 68]. These findings directly link TAD reorganization to cancer-relevant mRNA expression changes, potentially indicating a major mechanism for differential mRNA transcription in cancer cells. Although TAD organization in melanoma cells has not been comprehensively analyzed, it might display alterations similar to those in other cancer types. This hypothesis is already supported by the fact that the chromatin modifier EZH2, which is commonly mutated or overexpressed in melanoma, has been shown to drive structural changes in chromatin domains in diffuse large B-cell lymphoma [69].

## Transcriptional regulators and alterations driving melanoma initiation and progression

**Fig. 2:**
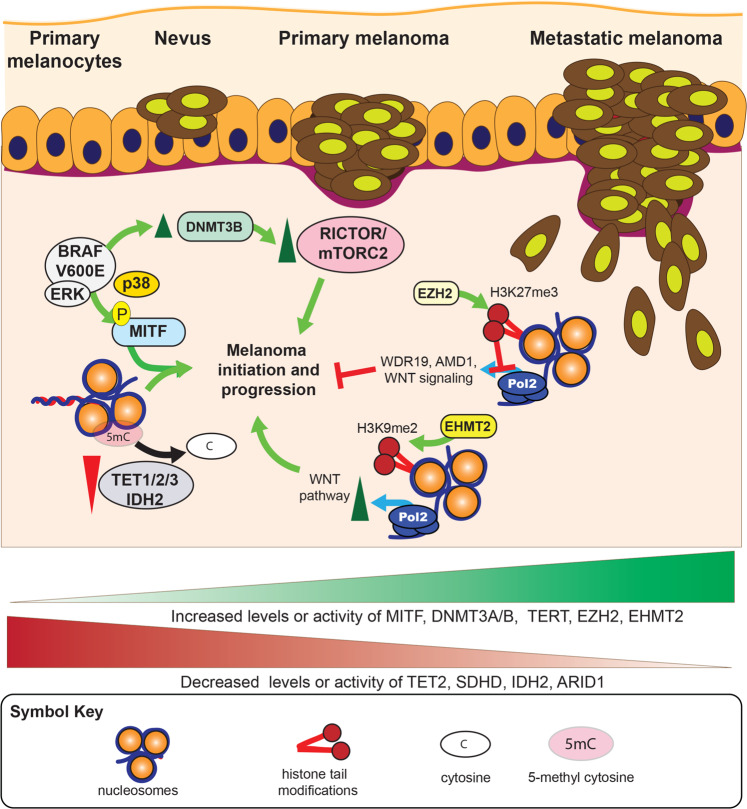
Functional impact of transcriptional mechanisms and regulators on various stages of melanoma initiation and progression. Several different transcriptional regulators and mechanisms play a direct functional role at various steps of melanoma initiation and progression in conjunction with other melanoma-associated alterations.

It is clear that a series of transcriptional regulators and mechanisms are dysregulated in melanoma, resulting in changes in mRNA expression and alteration of the melanoma proteome. However, the identification of alterations in transcription factors or chromatin regulators alone does not provide any information on the functional implications of these alterations in melanoma initiation and/or progression. Therefore, in most cases, further functional validation studies were conducted to establish the importance of discovered alterations in melanoma initiation and/or progression (Fig. 2). One example of a direct association between an altered transcription factor and melanoma initiation and progression is the transcription factor MITF. MITF is overexpressed in almost 20% of metastatic melanomas. The study that discovered MITF as an oncogene important for lineage survival showed that co-expression of oncogenic BRAF (BRAFV600E) and MITF resulted in the transformation of immortalized melanocytes [30]. Contrary to that, inhibition of MITF activity by expression of dominant-negative MITF in MITF-overexpressing melanoma blocked tumor growth, providing functional evidence of the role of MITF in melanoma initiation and tumor growth [30]. Further studies to identify the transcriptional targets of MITF in melanoma showed that MITF stimulates the expression of a number of cancer-promoting genes, including genes involved in cell cycle progression, differentiation, motility, and apoptosis [7].

In addition to direct transcription factor action in melanoma progression, DNA methylation-regulating proteins such as DNMTs and the TET family of active DNA demethylases have been shown to play an important role in melanoma initiation and progression [38, 40, 70]. One study showed that IDH2 and TET1 expression was reduced in melanoma compared with that in melanocytic naevi [40]. The same study showed that 5hmC levels were also low in melanoma cells, and restoration of 5hmC by ectopic expression of IDH2 or TET2 suppressed melanoma growth and increased tumor-free survival in animal models [40].

DNMT3B has also been shown to promote melanoma growth. The loss of Dnmt3b in a Braf/Pten mouse model resulted in a dramatic decrease in melanoma formation [38]. The authors of that study went on to show that the effect of Dnmt3b loss was due to the ability of Dnmt3b to repress miR-196b, which negatively regulates mTORC2 component Rictor. It is possible, however, that such a dramatic effect on melanoma development might be due to more than miR-196b regulation, and that protein-coding mRNAs might also play a role. In any case, the results clearly indicated that Dnmt3b is a pro-tumorigenic protein in melanoma and necessary for melanoma formation in the context of BRAF mutation and loss of phosphatase and tensin homolog (PTEN).

Another example of a functional association between mutation/overexpression and melanoma initiation and progression is the chromatin modifier EZH2. Several studies have shown an important role for EZH2 in melanoma initiation and progression [71–73]. All of these studies confirmed the occurrence of EZH2 mutation and overexpression in melanoma. One study showed that conditional Ezh2 ablation or inhibition with the EZH2 inhibitor GSK305 prevented melanoma progression in Tyr: NRAS^Q61K^Ink4a−/− mice [71]. The effect of Ezh2 depletion translated into almost full inhibition of lymph-node and distal lung metastases, which dramatically increased metastasis-free survival. The Ezh2 depletion did not affect normal melanocyte biology, however [71]. The same study identified functionally distinct suppressors of melanoma as targets of EZH2. For example, simultaneous knockdown of EZH2 and its target WDR19 reversed cell cycle arrest, whereas that of EZH2 and AMD1 restored the invasion capacity of melanoma cells without reversing the cell cycle-arrest phenotype [71]. A second study looked at the ability of the Ezh2^Y641F^ allele to cooperate with the conditionally activatable alleles Braf^V600E^ and Nras^Q61R^ in the presence of the tamoxifen-inducible tyrosinase-Cre allele (Tyr-Cre^ERT2^) in mice [72]. This study found that the Ezh2^Y641F^ allele cooperates with Braf^V600E^ alone or in combination with PTEN haploinsufficiency to promote melanoma formation and maintenance. However, the Ezh2^Y641F^ allele did not accelerate Nras^Q61R^ melanomagenesis, with or without the loss of p16^Ink4a^. Further analysis of previous sequencing results showed that EZH2 mutations in human melanoma co-occur with activating mutations in BRAF (*p* = 0.006) and are mutually exclusive with NRAS mutations (*p* = 0.004) [72]. In addition, the same study showed that Ezh2^Y641F^ expression caused global redistribution of H3K27me3. A more recent study showed that EZH2 plays an important role in melanoma genesis and metastasis by silencing genes that are necessary for the integrity of primary cilia [73]. This study went on to show that EZH2-mediated primary cilium disassembly enhances WNT/β-catenin signaling and promotes melanoma growth and metastasis. There were some differences in terms of the disease course and the mechanisms by which EZH2 promoted melanoma initiation and progression in the different studies [71–73]. Further studies are required to fully understand the reason for these differences. Nonetheless, all three studies concluded that EZH2 is an oncogenic protein of significance in melanoma that promotes melanoma initiation and progression.

Similar to EZH2, the histone methyltransferase EHMT2 drives melanoma growth and promotes an immunosuppressive microenvironment by activating the WNT signaling pathway. Melanoma cells with high levels of H3K9me2, a histone modification associated with EHMT2 enzymatic activity, were sensitive to the EHMT2 inhibitor UNC0642, indicating a dependency of EHMT2/G9a-amplified melanoma on EHMT2/G9a [51]. Furthermore, EHMT2 inhibition in melanoma resulted in increased immune cell-based tumor clearance, indicating a heightened immune response [51]. Together, these results indicated that EHMT2 drives tumorigenesis and a “cold” immune microenvironment by activating WNT signaling through DKK1 repression.

TERT promoter mutations are present in melanoma; however, it is not yet known how they function to promote melanoma. One possibility is that TERT promoter mutations drive increased TERT expression and increased telomerase activity. The treatment of melanoma cells with BRAF inhibitors reduced TERT expression and telomerase activity, suggesting that the MAPK pathway was necessary for TERT expression in these cells [74]. The same study showed that TERT ectopic expression alone was sufficient to rescue the growth of melanoma cells expressing BRAF-targeting short hairpin RNA. These results highlight the importance of TERT gene expression in melanoma; however, more detailed studies are required to determine the exact impact of TERT promoter mutations in melanoma.

## Transcriptional deregulation as a targetable melanoma liability

**Fig. 3:**
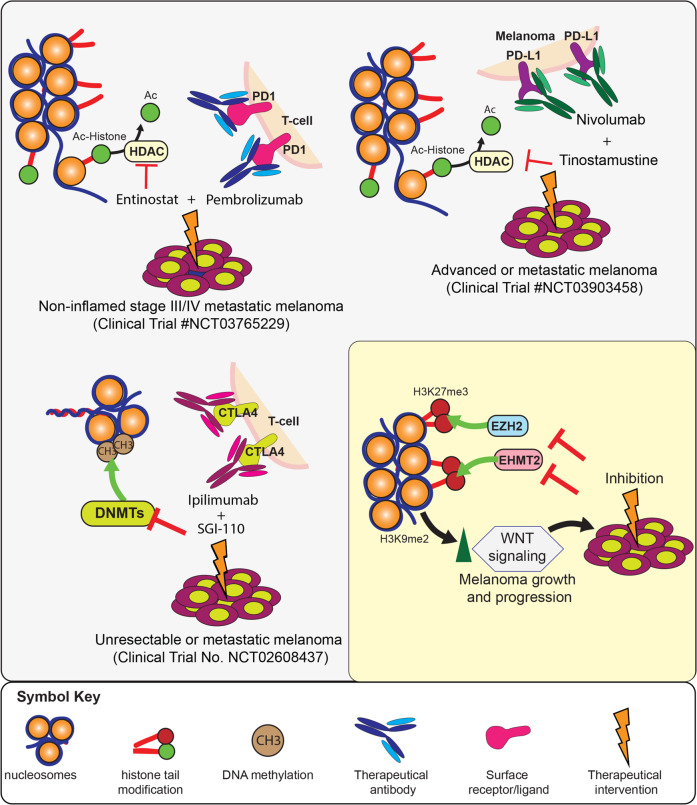
Therapeutic targeting of transcriptional deregulation for melanoma therapy. A series of drug combinations with DNMT and HDAC inhibitors are undergoing clinical trials for their utility in the treatment of metastatic melanoma. Many other proteins, such as EZH2, have shown promise in clinical trials in some cancers and, on the basis of functional studies in melanoma cells, are possible targets for clinical development. Similarly, there are a group of targets, such as EHMT2/G9a, which are not yet in clinical trials, but preclinical data make them strong drug candidates for metastatic melanoma therapy.

Melanoma is arguably at the top of the list in regard to successful outcomes achieved with next-generation personalized therapies and immunotherapies. However, not all melanoma patients benefit from BRAF/MEK inhibitors or immunotherapies. Therefore, the quest to identify additional druggable targets in melanoma is still ongoing. Chromatin modifiers and transcription factors such as BCL6 have emerged as potential drug targets in various cancers [75, 76]. There are currently several ongoing clinical trials utilizing different histone deacetylation inhibitors with other therapeutic agents to treat metastatic melanoma (Fig. 3). One example is the use of the class I histone deacetylase (HDAC) inhibitor entinostate in combination with pembrolizumab (Keytruda) to treat noninflamed stage III/IV metastatic melanoma (Clinical Trial No. NCT03765229). Pembrolizumab is a highly selective humanized monoclonal IgG4 antibody directed against the PD-1 receptor on the surface of T-cells. The idea behind the combination of pembrolizumab and entinostate is that treatment with entinostate will sensitize metastatic melanoma cells that are otherwise resistant to pembrolizumab by causing T-cell infiltration in noninflamed melanoma. Another drug combination that is under investigation is tinostamustine and the anti-PD-L1 antibody nivolumab (OPDIVO) (Clinical Trial No. NCT03903458). Tinostamustine is an alkylating histone deacetylase inhibitor (HDACi) fusion molecule composed of the alkylating agent bendamustine fused to the pan-HDACi vorinostat. It is hoped that the combination will exert an anti-neoplastic effect by enhancing the efficacy of nivolumab. A similar example is a clinical study investigating the use of SGI-110 in combination with the anti-CTLA4 antibody ipilimumab (Yervoy) to treat unresectable or metastatic melanoma (Clinical Trial No. NCT02608437). SGI-110 is a DNA methyltransferase inhibitor whose active metabolite is the U.S. Food and Drug Administration-approved drug decitabine. There is preclinical evidence that SGI-110 has an immunomodulatory effect, providing the basis for the combination trial with ipilimumab. The outcomes of the current clinical trials will further guide the clinical use of drugs targeting chromatin and DNA modifiers and will hopefully provide new therapeutic options for patients with metastatic melanoma.

## Conclusion and future prospects

Although it is clear that melanoma is highly dependent on transcriptional regulators for initiation and progression, the clinical use of transcriptional regulatory proteins in patients with melanoma is restricted compared with that in patients with other types of cancers, and is thus far largely restricted to HDAC inhibitors and DNMT inhibitors. The substantial activity in the development of clinical drugs targeting transcriptional regulatory proteins in other types of cancers might provide significant clinical opportunities for the treatment of metastatic melanomas that are refractory to currently approved therapies. One major advantage of pursing that line of inquiry is the fact that for several clinical grade inhibitors, such as EZH2 and embryonic ectoderm development (EED) proteins, data on human pharmacokinetics, pharmacodynamics, and toxicity and, in some cases, clinical trial outcomes in other cancer types are already available [77]. Therefore, it would be relatively easy to set up informed clinical trials using these inhibitors to treat melanoma. The list of transcriptional regulators that melanoma cells depend on for survival is growing. These regulators have the potential to be used to treat all subtypes of melanoma because many of the dependencies extend beyond the BRAF-mutant subtype of melanoma. Future studies in preclinical and clinical settings will hopefully identify new drug targets with widespread utility against metastatic melanoma in patient populations for which current therapies either do not work or to which resistance has emerged.
